# Comparative effectiveness of adjunctive rifampicin versus gentamicin for prosthetic valve endocarditis due to *Staphylococcus aureus*

**DOI:** 10.1093/jacamr/dlaf246

**Published:** 2025-12-16

**Authors:** Taito Kitano, Sayaka Yoshida

**Affiliations:** Department of Paediatrics, Nara Prefecture General Medical Centre, Nara, Japan; Clinical Research Centre, Nara Prefecture General Medical Centre, Nara, Japan; Department of Paediatrics, Nara Prefecture General Medical Centre, Nara, Japan; Clinical Research Centre, Nara Prefecture General Medical Centre, Nara, Japan

## Abstract

**Background:**

Although adjunctive rifampicin and/or gentamicin have been recommended for *Staphylococcus aureus* prosthetic valve endocarditis, evidence regarding the evaluation of their clinical effectiveness is limited.

**Objectives:**

To compare the clinical impact of adjunctive rifampicin without gentamicin, and adjunctive gentamicin without rifampicin therapies for *S. aureus* prosthetic valve endocarditis.

**Methods:**

This retrospective study used TriNetX to evaluate multicentre electronic medical records of patients aged 18 years or older in the USA between 2016 and 2024. After propensity score matching, HRs were estimated with 95% CIs. Covariates included age, sex, ethnicity and medical comorbidities.

**Results:**

A total of 353 and 369 patients were identified in the rifampicin and gentamicin groups, respectively. One-year all-cause mortality was observed in 87 (31.3%) and 111 (39.9%) patients in the rifampicin and gentamicin groups after propensity score matching, respectively, leading to an HR of 0.71 (95% CI, 0.54–0.94; *P* = 0.016). The HRs were not statistically significant for ICU admission (HR 0.93; 95% CI, 0.74–1.18; *P* = 0.540), recurrent endocarditis (HR 0.76; 95% CI, 0.42–1.40; *P* = 0.381), kidney failure (HR 0.93; 95% CI, 0.74–1.18; *P* = 0.540) or hepatic failure (HR 0.96; 95% CI, 0.66–1.39; *P* = 0.822).

**Conclusions:**

The rifampicin-containing regimen without gentamicin was associated with reduced 1 year mortality compared with the gentamicin-containing regimen without rifampicin. Although the results should be interpreted with caution because of potential residual unmeasured confounders, including duration of antimicrobial treatment and biases, our findings provide further evidence that adjunctive gentamicin may not be routinely needed for *S. aureus* prosthetic valve endocarditis.

## Introduction

Prosthetic valve endocarditis is a severe infectious disease associated with high mortality, with a 1 year mortality rate of over 30%, irrespective of its causal pathogen.^1^  *Staphylococcus aureus* is the most common cause of prosthetic valve endocarditis,^1,2^ and is associated with higher mortality in infective and prosthetic valve endocarditis compared with other causal pathogens.^1,3,4^ Given the high mortality of *S. aureus* prosthetic valve endocarditis, determining the best therapeutic option, including the choice of combination antimicrobial therapies, is crucial.

Combination therapy with two or more antimicrobials has been frequently implemented for prosthetic valve endocarditis.^5^ For *S. aureus* prosthetic valve endocarditis, the European Society of Cardiology guideline recommends rifampicin and gentamicin as adjunctive therapy.^6^ The 2015 guideline from the American Heart Association also recommends the addition of rifampicin and gentamicin.^7^ Another recent guideline removed gentamicin from the treatment options for *S. aureus* prosthetic valve endocarditis.^8^ Thus, whether gentamicin should be added to the treatment of *S. aureus* prosthetic valve endocarditis remains controversial.^9^ Although the recommendation of gentamicin therapy is mainly based on preclinical studies and case reports, as well as the high mortality associated with the disease, there is little evidence regarding the clinical effectiveness of adjunctive rifampicin and gentamicin.^9,10^

Establishing evidence for the comparative effectiveness of adjunctive rifampicin versus gentamicin may help guide standardized combination therapy for *S. aureus* prosthetic valve endocarditis. A retrospective study did not find that adjunctive gentamicin or rifampicin was associated with improved clinical outcomes in 373 patients with staphylococcal prosthetic valve endocarditis, including *S. aureus* and other staphylococci.^11^ A systematic review and meta-analysis of the clinical impact of adding gentamicin and rifampicin for staphylococcal prosthetic valve endocarditis, including both *S. aureus* and other staphylococci, identified only four studies: two of these indicated no clinical improvement after adding gentamicin to a rifampicin-containing regimen, and two reported no clinical benefits of adding rifampicin to a gentamicin-containing regimen.^12^ Thus, there are some clinical studies comparing gentamicin plus rifampicin with gentamicin or rifampicin alone. However, none of the identified studies compared rifampicin-containing regimens without gentamicin with gentamicin-containing regimens without rifampicin. In addition, although these previously published studies did not show the clinical benefits of adding gentamicin, they may have lacked sufficient power to detect a significant difference because of their relatively small sample sizes. Furthermore, these studies combined data from both *S. aureus* and other staphylococci (i.e. CoNS). As the clinical courses of *S. aureus* and other staphylococci are generally expected to differ,^13,14^ the clinical impact of adjunctive therapy for *S. aureus* prosthetic valve endocarditis should be distinguished from that for other staphylococcal infections.

This study aimed to compare the clinical impact of adjunctive rifampicin without gentamicin with adjunctive gentamicin without rifampicin therapies for *S. aureus* prosthetic valve endocarditis.

## Patients and methods

### Ethics

The TriNetX database used in this study complies with data privacy regulations, including Health Insurance Portability and Accountability Act (HIPAA) regulations, and is certified by ISO 27001:2013. All data in the TriNetX platform are deidentified according to HIPAA standards.^15^ Therefore, studies using the TriNetX platform do not require institutional review board approval, consistent with the stipulations of the basic Health and Human Services policy for the protection of human research subjects. However, the Institutional Review Board of Nara Prefecture General Medical Centre reviewed the study and approved its publication (Approval No, 1050).

### Study design and population

This retrospective study aimed to compare the clinical prognoses of *S. aureus* prosthetic valve endocarditis following adjunctive rifampicin therapy without gentamicin with adjunctive gentamicin therapy without rifampicin in the USA. We used TriNetX, a multicentre electronic medical records database that connects 72 US healthcare organizations as of October 2025.^15^ TriNetX contains patients’ medical records, including diagnoses, medications, procedures, laboratory tests and clinical encounters. Within the TriNetX database, researchers with data access can create two cohorts to conduct propensity score-based outcome comparisons. The data search period was from January 2016 to October 2024 (to allow a sufficient outcome follow-up period).

Eligible participants were those aged 18 years or older diagnosed with prosthetic valve endocarditis (T82.6: infection and inflammatory reaction due to cardiac valve prosthesis) based on ICD-10, and *S. aureus* infection. The two cohorts consisted of patients who received rifampicin without gentamicin (rifampicin group) and those who received gentamicin without rifampicin (gentamicin group) within 7 days of diagnosis (Tables S1 and S2, available as Supplementary data at *JAC-AMR* Online). The date of diagnosis was based on appearance of ICD-10 coding. A previous study showed that ICD-10 coding could accurately identify endocarditis (sensitivity 90%–95% and specificity 100%).^16^ The primary outcome was 1 year all-cause mortality, and secondary outcomes included critical care admission, recurrent endocarditis, kidney failure (ICD-10 code N17–19) and hepatic failure or toxic liver injury (ICD-10 code K71–72). Outcomes were followed up for 1 year from the day after diagnosis, except for recurrent endocarditis, which was followed between Day 43 (after standard treatment duration for prosthetic valve endocarditis) and Day 365 since the first diagnosis of prosthetic valve endocarditis. Participants’ medical comorbidities were also extracted based on ICD-10 codes (Table 1).

**Table 1.** Participants’ characteristics before propensity score matching between rifampicin group and gentamicin group

	Rifampicin group (*n* = 353)	Gentamicin group (*n* = 369)	*P* value
Age group, y			
18–49	125 (35.4%)	164 (44.4%)	0.013
50–64	77 (21.8%)	98 (26.6%)	0.137
65–79	118 (33.4%)	85 (23.0%)	0.002
80 or older	33 (9.3%)	22 (6.0%)	0.086
Gender			
Female	114 (32.3%)	139 (37.7%)	0.130
Race/ethnicity			
White	306 (86.7%)	296 (80.2%)	0.020
Black or African American	14 (4.0%)	45 (12.2%)	<0.001
Asian	≤10 (NA)	≤10 (NA)	NA
Others	≤10 (NA)	≤10 (NA)	NA
Unknown	22 (6.2%)	13 (3.5%)	0.090
Comorbidity			
Presence of cardiac pacemaker (Z95.0)	55 (15.6%)	60 (16.3%)	0.803
Presence of other cardiac and vascular implants and grafts (Z95.8)	56 (15.9%)	61 (16.5%)	0.808
Mental and behavioural disorders due to psychoactive substance use (F10–19)	131 (37.1%)	184 (49.9%)	0.001
Heart failure (I50)	199 (56.4%)	224 (60.7%)	0.238
Diabetes mellitus (E08-13)	113 (32.0%)	119 (32.2%)	0.945
Certain disorders involving the immune mechanism (D80-89)	13 (3.7%)	25 (6.8%)	0.063
Chronic lower respiratory diseases (J40-4A)	91 (25.8%)	110 (29.8%)	0.227
Diseases of the nervous system (G00-99)	237 (67.1%)	285 (77.2%)	0.002
Acute kidney failure and chronic kidney disease (N17-19)	237 (67.1%)	277 (75.1%)	0.019
Shock, not elsewhere classified (R57)	66 (18.7%)	99 (26.8%)	0.009
Methicillin-resistant *Staphylococcus aureus* infection, unspecified site (A49.02)	21 (5.9%)	28 (7.6%)	0.381
Methicillin-resistant *Staphylococcus aureus* infection as the cause of diseases classified elsewhere (B95.62)	87 (24.6%)	96 (26.0%)	0.672
Sepsis due to methicillin-resistant *Staphylococcus aureus* (A41.02)	76 (21.5%)	94 (25.5%)	0.212
History of ICU admission	142 (40.2%)	200 (54.2%)	<0.001
Administered antimicrobials			
Cefazolin	127 (36.0%)	124 (33.6%)	0.586
Anti-staphylococcal (MSSA) penicillins	91 (25.8%)	31 (8.4%)	<0.001
Vancomycin	198 (56.1%)	284 (77.0%)	<0.001
Daptomycin	56 (15.9%)	55 (14.9%)	0.757
Linezolid	14 (4.0%)	≤10 (≤2.7%)	NA
Ceftaroline	26 (7.4%)	40 (10.8%)	0.121

NA, not assessed.

Comorbidity was based on ICD-10 codes provided on the day of prosthetic valve endocarditis diagnosis or before.

### Statistical analysis

Propensity score matching was performed to balance covariates. Covariates included in the propensity score matching were patient age group, sex, ethnicity, medical comorbidities, including heart failure, presence of an intracardiac device or pacemaker, diabetes mellitus, immunocompromising conditions, chronic lower respiratory diseases, neurological diseases, acute kidney failure or chronic kidney disease, substance abuse, presence of MRSA infection, presence of shock, and a history of ICU admission, as reported in previous studies evaluating risk factors for severe outcomes following prosthetic valve endocarditis or infective endocarditis.^3,17–22^ The risk ratio (RR) with a 95% CI was calculated by dividing the risk in the exposure group (e.g. rifampicin group) by the risk in the comparison group (e.g. gentamicin group). Chi-square tests were used to compare categorical outcomes. HR with 95% CI was estimated using the Cox proportional hazard model (R 4.0.2, Survival package 3.2-3, embedded in the TriNetX platform). The log-rank test was performed to compare survival curves.

Subgroup analyses were conducted by age group (young adults aged 18–64 years versus elderly individuals aged 65 years or older), diagnosis of MSSA versus MRSA, and right-sided versus left-sided endocarditis. A sensitivity analysis was conducted by including only early prosthetic valve endocarditis (<12 months between valve surgery and the onset of prosthetic valve endocarditis) and by limiting outcome follow-up period to 90 days.^23^ Given that some current guidelines recommend both rifampicin and gentamicin, additional analyses were conducted by comparing the rifampicin group with the rifampicin plus gentamicin group, and by comparing the gentamicin group with the rifampicin plus gentamicin group. All analyses were conducted using the TriNetX database. The propensity scores were matched for each analysis.

## Results

A total of 353 and 369 patients were identified in the rifampicin and gentamicin groups, respectively. The patient characteristics of the two groups are presented in Table 1. Prior to propensity score matching, the rifampicin group was older than the gentamicin group (35.3% and 44.4% aged 18–49 years, *P* = 0.013; 33.4% versus 23.0% aged 65–79 years or older, *P* = 0.002). In the rifampicin and gentamicin groups, 32.3% and 37.7% of the patients, respectively, were female. A higher proportion of participants in the rifampicin group were Caucasian (86.7% versus 80.2%; *P* = 0.020), whereas the gentamicin group had a higher proportion of Black and African American participants (4.0% versus 12.2%; *P* < 0.001). The proportions of participants with mental and behavioural disorders due to psychoactive substance use (37.1% and 49.9%; *P* = 0.001), kidney disease (67.1% and 75.1%; *P* = 0.019), nervous system diseases (67.1% and 77.2%; *P* = 0.002), and a history of ICU admission (40.2% and 54.2%; *P* < 0.001) were higher in the gentamicin group.

**Figure 1.**
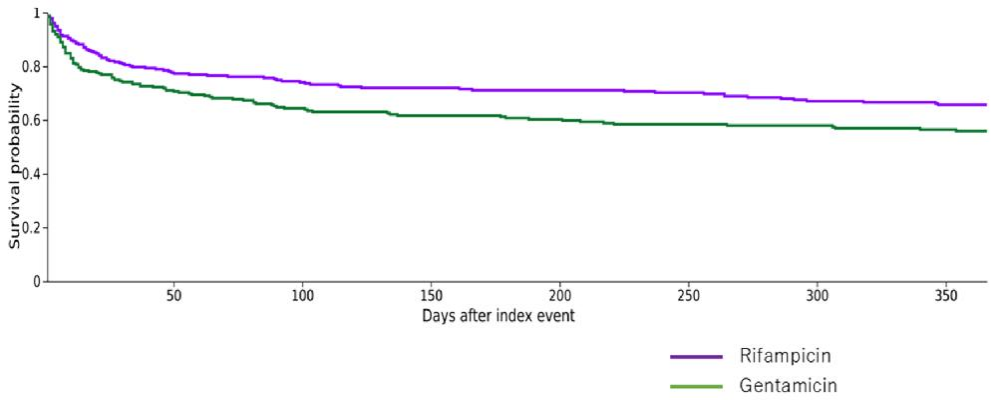
Survival curve in rifampicin group versus gentamicin group since diagnosis (index event).

After propensity score matching, 278 participants from each group remained, and residual standard differences were below 0.1 for all included covariates (Table 2). The primary outcome, 1 year all-cause mortality, was observed in 87 (31.2%) and 111 (39.9%) patients in the rifampicin and gentamicin groups after the propensity score matching, respectively, leading to an HR of 0.71 (95% CI, 0.54–0.94; *P* = 0.016) (Figure 1). For secondary outcomes, the HRs were not statistically significant for ICU admission (HR 0.93; 95% CI, 0.74–1.18; *P* = 0.540), recurrent endocarditis (HR 0.76; 95% CI, 0.42–1.40; *P* = 0.381), kidney failure (HR 0.93; 95% CI, 0.74–1.18; *P* = 0.540), or hepatic failure (HR 0.96; 95% CI, 0.66–1.39; *P* = 0.822). The trends in statistical significance for the primary and secondary outcomes were similar for the RR (Table 3).

**Table 2.** Participants’ characteristics after propensity score matching between rifampicin group and gentamicin group

	Rifampicin group (*n* = 278)	Gentamicin group (*n* = 278)	*P* value	Standard difference
Age group, y				
18–49	111 (39.9%)	112 (40.3%)	0.931	0.007
50–64	71 (25.5%)	65 (23.4%)	0.554	0.050
65–79	74 (26.6%)	79 (28.4%)	0.635	0.040
80 years or older	22 (7.9%)	22 (7.9%)	1.000	<0.001
Gender				
Female	94 (33.8%)	95 (34.2%)	0.929	0.008
Race/ethnicity				
White	236 (84.9%)	241 (86.7%)	0.544	0.052
Black or African American	14 (5.0%)	12 (4.3%)	0.688	0.034
Asian	≤10 (NA)	≤10 (NA)	NA	NA
Others	≤10 (NA)	≤10 (NA)	NA	NA
Unknown	19 (6.8%)	13 (4.7%)	0.275	0.093
Comorbidity				
Presence of cardiac pacemaker (Z95.0)	46 (16.5%)	47 (16.9%)	0.910	0.010
Presence of other cardiac and vascular implants and grafts (Z95.8)	45 (16.2%)	45 (16.2%)	1.000	<0.001
Mental and behavioural disorders due to psychoactive substance use (F10-19)	115 (41.4%)	124 (44.6%)	0.441	0.065
Heart failure (I50)	160 (57.6%)	163 (58.6%)	0.797	0.022
Diabetes mellitus (E08-13)	88 (31.7%)	86 (30.9%)	0.855	0.016
Certain disorders involving the immune mechanism (D80-89)	12 (4.3%)	13 (4.7%)	0.838	0.017
Chronic lower respiratory diseases (J40-4A)	81 (29.1%)	79 (28.4%)	0.851	0.016
Diseases of the nervous system (G00-99)	198 (71.2%)	202 (72.7%)	0.706	0.032
Acute kidney failure and chronic kidney disease (N17-19)	196 (70.5%)	202 (72.7%)	0.573	0.048
Shock, not elsewhere classified (R57)	59 (21.2%)	62 (22.3%)	0.758	0.026
Methicillin-resistant *Staphylococcus aureus* infection, unspecified site (A49.02)	18 (6.5%)	21 (7.6%)	0.618	0.042
Methicillin-resistant *Staphylococcus aureus* infection as the cause of diseases classified elsewhere (B95.62)	68 (24.5%)	70 (25.2%)	0.844	0.017
Sepsis due to methicillin-resistant *Staphylococcus aureus* (A41.02)	63 (22.7%)	65 (23.4%)	0.840	0.017
History of ICU admission	129 (46.4%)	120 (43.2%)	0.443	0.065

NA, not assessed.

Comorbidity was based on ICD-10 codes provided on the day of prosthetic valve endocarditis diagnosis or before.

**Table 3.** Primary and secondary outcomes for participants overall after propensity score matching

	Rifampicin group (*n* = 278)	Gentamicin group (*n* = 278)	RR [95%CI]	*P* values for RR	HR [95%CI]	*P* values for HR
Primary outcome						
All-cause mortality	87 (31.3%)	111 (39.9%)	0.78 [0.63, 0.98]	0.034	0.71 [0.54, 0.94]	0.016
Secondary outcome						
ICU admission	137 (49.3%)	143 (51.4%)	0.96 [0.81, 1.13]	0.611	0.93 [0.74, 1.18]	0.540
Recurrent endocarditis^a^	19 (6.8%)	23 (8.3%)	0.83 [0.46, 1.48]	0.521	0.76 [0.42, 1.40]	0.381
Renal failure	137 (49.3%)	143 (51.4%)	0.96 [0.81, 1.13]	0.611	0.93 [0.74, 1.18]	0.540
Hepatic failure	57 (20.5%)	54 (19.4%)	1.06 [0.76, 1.47]	0.750	0.96 [0.66, 1.39]	0.822

PVE, prosthetic valve endocarditis; RR, risk ratio.

Outcomes were followed for 1 year since the diagnosis of prosthetic valve endocarditis.

^a^Recurrent endocarditis was another diagnosis of T82.6 in ICD-10 code between Day 43 (after standard treatment duration for PVE) and Day 365 since the first diagnosis of PVE.

In the subgroup analyses, the 1 year all-cause mortality rate was not significantly different in younger adults (HR 0.79; 95% CI, 0.35–1.76; *P* = 0.559), the elderly (HR 0.90; 95% CI, 0.58–1.38; *P* = 0.617), MSSA diagnosis (HR 0.93; 95% CI, 0.66–1.32; *P* = 0.688), right-sided endocarditis (HR 0.66; 95% CI, 0.31–1.40; *P* = 0.273) or left-sided endocarditis (HR 0.73; 95% CI, 0.51–1.06; *P* = 0.099). The HR of 1 year all-cause mortality was 0.63 (95% CI, 0.39–0.99; *P* = 0.044) for MRSA diagnosis. In the sensitivity analysis, limiting to early prosthetic valve endocarditis showed no significant difference in 1 year all-cause mortality (RR 0.93; 95% CI, 0.43–2.01; *P* = 0.470). Limiting the outcome follow-up period to 90 days maintained a statistical reduction of all-cause mortality (HR 0.66; 95% CI, 0.47–0.91; *P* = 0.010). The HRs of 1 year all-cause mortality were 0.97 (95% CI, 0.74–1.27; *P* = 0.842) and 1.32 (95% CI, 1.03–1.70; *P* = 0.027) in the rifampicin group versus the rifampicin plus gentamicin group, and in the gentamicin group versus the rifampicin plus gentamicin group, respectively (Tables S3–S5, Figures S1 and S2). These statistical trends were similar for the RR (Table 4).

**Table 4.** Subgroup and sensitivity analyses for primary outcome after propensity score matching

	RR [95%CI]	*P* values for RR	HR [95%CI]	*P* values for HR
Rifampicin versus gentamicin (*n* = 278)	0.78 [0.63, 0.98]	0.034	0.71 [0.54, 0.94]	0.016
Subgroup analysis				
By age group				
18–64 years (*n* = 163)	0.85 [0.39, 1.83]	0.671	0.79 [0.35, 1.76]	0.559
65 years or older (*n* = 92)	0.95 [0.69, 1.32]	0.767	0.90 [0.58, 1.38]	0.617
By diagnosis of MSSA versus MRSA				
MSSA (*n* = 173)	1.00 [0.76, 1.00]	1	0.93 [0.66, 1.32]	0.688
MRSA (*n* = 98)	0.72 [0.50, 1.04]	0.077	0.63 [0.39, 0.99]	0.044
By right- versus left-sided PVE				
Right-sided (*n* = 42)	0.75 [0.41, 1.39]	0.355	0.66 [0.31, 1.40]	0.273
Left-sided (*n* = 130)	0.81 [0.61, 1.07]	0.134	0.73 [0.51, 1.06]	0.099
Sensitivity analysis				
Early PVE only (*n* = 37)	1.00 [0.54, 1.86]	1	0.93 [0.43, 2.01]	0.470
Outcome follow-up period 90 days (*n* = 263)	0.72 [0.54, 0.94]	0.016	0.66 [0.47, 0.91]	0.010
Rifampicin versus rifampicin plus gentamicin (*n* = 324)	1.02 [0.82, 1.27]	0.867	0.97 [0.74, 1.27]	0.842
Gentamicin versus rifampicin plus gentamicin (*n* = 347)	1.22 [1.00, 1.50]	0.048	1.32 [1.03, 1.70]	0.027

PVE, prosthetic valve endocarditis.

## Discussion

Some guidelines recommend addition of rifampicin plus gentamicin for treatment of prosthetic valve endocarditis.^6,7^ However, the role of this combination therapy has been questioned by recent clinical data.^11,12^ Our study compared the clinical outcomes of *S. aureus* prosthetic valve endocarditis treated with rifampicin-containing therapy without gentamicin versus gentamicin-containing therapy without rifampicin. In the overall study population, a significantly lower 1 year all-cause mortality rate was observed in the rifampicin group than in the gentamicin group, suggesting rifampicin may be superior to gentamicin. Secondary outcomes, including ICU admission as well as renal and hepatic failure, were not statistically different between the two groups. The lack of statistical differences in the secondary outcomes may reflect limited statistical power and the low incidence of some outcome events (e.g. renal and hepatic failures). In addition, although rifampicin and gentamicin are known to be associated with adverse hepatic and renal events, some clinicians may avoid their use in patients at high risk of developing hepatic or renal failure. These considerations indicate that our findings for the secondary outcomes should be interpreted with caution.

This study is among the first to demonstrate the superiority of a rifampicin-containing regimen without gentamicin over a gentamicin-containing regimen without rifampicin. The increased mortality observed in the gentamicin group supports the use of adjunctive rifampicin instead of gentamicin for prosthetic valve endocarditis, unless there is a specific reason to prefer gentamicin. Although some current guidelines still recommend adjunctive gentamicin therapy with rifampicin,^6,7^ our study provides evidence that adjunctive gentamicin may not be needed for prosthetic valve endocarditis. Other studies also questioned the role of adding gentamicin for prosthetic valve endocarditis.^11,12^ The recent evidence, including the results of this study, may affect the recommendation of adding gentamicin in *S. aureus* prosthetic valve endocarditis in future guidelines. Other large-scale studies with similar designs using different databases as well as comparisons between adjunctive rifampicin without gentamicin and no adjunctive therapy may further strengthen the evidence. Given the nature of observational studies, the possibility of immortal-time bias should always be considered.

In the subgroup and sensitivity analyses, no statistically significant differences in mortality were observed, except for MRSA endocarditis. This could be because of the relatively small sample size, which divided the overall study population. The reducing trend of mortality in the rifampicin group in MRSA diagnosis may have been associated with rifampicin’s coverage of MRSA, which is not covered by some primary antimicrobials (e.g. cefazolin and oxacillin), compared with MSSA, which is usually covered by primary antimicrobials.^24^ In addition, we relied on ICD-10 codes for the status identification of MSSA versus MRSA diagnoses and early prosthetic valve endocarditis. This may have led to an underestimation of the identified cases.^25,26^

The advantages of this study included the use of the large-scale database, which enabled clinical comparisons between the rifampicin and gentamicin groups. Given that the numbers of patients with *S. aureus* prosthetic valve endocarditis who received rifampicin without gentamicin or those who received gentamicin without rifampicin were less compared with those who received both rifampicin and gentamicin, a large database was required to conduct statistical analyses.

However, this study has several limitations. First, we did not have access to individual patient-level data in the TriNetX. Compared with previous relevant studies with individual patient-level data,^11,27,28^ the possibility of introducing bias, including case ascertainment bias, should be considered in this retrospective study without adjudication.^29^ The potential ascertainment bias could be applied to both rifampicin and gentamicin groups. Although a previous study showed that ICD-10 coding could accurately identify endocarditis,^15^ whether this can be applied to prosthetic valve endocarditis has not been extensively investigated. In addition, this study is subject to immortal-time bias. Second, we could not evaluate the impact of participating healthcare organizations (e.g. large versus small centres, or urban versus rural settings). Third, we could not evaluate the potential impact of unmeasured confounders, as factors that were not included in our analyses may have influenced the results. Fourth, the sample sizes in the subgroup and sensitivity analyses were relatively small; therefore, future studies with a larger sample size may help to investigate further the potential differential impact of rifampicin versus gentamicin at a subpopulation level. Fifth, because microbiological data were rarely available in the TriNetX database, we could not evaluate potentially important microbiological factors, including antimicrobial sensitivity. For example, we relied on ICD-10-based diagnosis of MSSA versus MRSA. Finally, we could not evaluate the impact of the main or other adjunctive antimicrobials (e.g. cefazolin or cloxacillin for MSSA, and vancomycin, daptomycin, linezolid or ceftaroline for MRSA) or antimicrobial duration in this study. Although evaluating multiple antimicrobial regimens and different durations makes the analyses more complex, future studies may include the potential differential impact of these regimens on *S. aureus* prosthetic valve endocarditis. Data regarding the need for antimicrobial discontinuation were not available in this study.

In conclusion, our study showed that a rifampicin-containing regimen without gentamicin was associated with reduced 1 year mortality compared with a gentamicin-containing regimen without rifampicin. Although these findings should be interpreted with caution because of the nature of the real-world data and potential residual unmeasured confounders, they provide further evidence that adjunctive gentamicin may not be routinely needed for *S. aureus* prosthetic valve endocarditis. Additional studies are warranted to confirm the clinical impact, as well as to explore the potential differential impact in subpopulations.

## Supplementary Material

dlaf246_Supplementary_Data

## Data Availability

The data supporting the findings of this study are available from TriNetX Network (https://trinetx.com). Individual-level data are not available because of TriNetX regulations. Additional analyses are available on request from the corresponding author. Although individual patient data from the TriNetX Network cannot be accessed to protect patient health information, researchers can apply for aggregate data access through the TriNetX Network.

## Supplementary data

Tables S1 to S5 and Figures S1 and S2 are available as Supplementary data at *JAC-AMR* Online.
