# Supplementary material for: Comparative effectiveness of adjunctive rifampicin versus gentamicin for prosthetic valve endocarditis due to Staphylococcus aureus
Source: JAC Antimicrob Resist. 2025 Dec 16;7(6):dlaf246. doi: 10.1093/jacamr/dlaf246 (PMC12706467; doi:10.1093/jacamr/dlaf246)

Supplemental Table 1. Extraction terms for rifampicin group.

| Group 1 | | | | | |
| --- | --- | --- | --- | --- | --- |
|  | **Group 1A** | | | | |
|  | must have |  | diagnosis | UMLS:ICD10CM:T82.6 | Infection and inflammatory reaction due to cardiac valve prosthesis (at least 18 years old at event) |
|  | date constraint | | The terms in this group occurred between Jan 1, 2016 and Oct 31, 2024 | | |
|  | event relationship | | Any instance of Group 1B occurred within 7 days on or after the first instance of Group 1A | | |
|  | **Group 1B** | | | | |
|  | must have |  | medication | NLM:RXNORM:9384 | rifampin |
|  | cannot have |  | medication | NLM:RXNORM:1596450 | gentamicin |
|  |  | or | medication | NLM:RXNORM:10627 | tobramycin |
|  |  | or | medication | NLM:RXNORM:641 | amikacin |
| Group 2 | | | | | |
|  | **Group 2A** | | | | |
|  | must have |  | diagnosis | UMLS:ICD10CM:T82.6 | Infection and inflammatory reaction due to cardiac valve prosthesis (at least 18 years old at event) |
|  | date constraint | | The terms in this group occurred between Jan 1, 2016 and Oct 31, 2024 | | |
|  | event relationship | | Any instance of Group 2B occurred within 7 days before or up to 7 days after the first instance of Group 2A | | |
|  | **Group 2B** | | | | |
|  | must have | any of | diagnosis | UMLS:ICD10CM:B95.61 | Methicillin susceptible Staphylococcus aureus infection as the cause of diseases classified elsewhere |
|  |  |  | diagnosis | UMLS:ICD10CM:A49.01 | Methicillin susceptible Staphylococcus aureus infection, unspecified site |
|  |  |  | diagnosis | UMLS:ICD10CM:A41.01 | Sepsis due to Methicillin susceptible Staphylococcus aureus |
|  |  |  | diagnosis | UMLS:ICD10CM:B95.62 | Methicillin resistant Staphylococcus aureus infection as the cause of diseases classified elsewhere |
|  |  |  | diagnosis | UMLS:ICD10CM:A49.02 | Methicillin resistant Staphylococcus aureus infection, unspecified site |
|  |  |  | diagnosis | UMLS:ICD10CM:A41.02 | Sepsis due to Methicillin resistant Staphylococcus aureus |
|  |  |  | diagnosis | UMLS:ICD10CM:B95.6 | Staphylococcus aureus as the cause of diseases classified elsewhere |
|  |  |  | diagnosis | UMLS:ICD10CM:A41.0 | Sepsis due to Staphylococcus aureus |
|  |  |  | laboratory | UMLS:LNC:88269-6 | Staphylococcus aureus gyrB gene [Presence] by Probe in Positive blood culture (labResult: Positive) |
|  |  |  | laboratory | UMLS:LNC:92777-2 | Staphylococcus aureus DNA [Presence] by NAA with probe detection in Positive blood culture (labResult: Positive) |
|  |  |  | laboratory | UMLS:LNC:85765-6 | Staphylococcus aureus DNA [Presence] by NAA with non-probe detection in Positive blood culture (labResult: Positive) |

Supplemental Table 2. Extraction terms for gentamicin group.

| Group 1 | | | | | |
| --- | --- | --- | --- | --- | --- |
|  | **Group 1A** | | | | |
|  | must have |  | diagnosis | UMLS:ICD10CM:T82.6 | Infection and inflammatory reaction due to cardiac valve prosthesis (at least 18 years old at event) |
|  | date constraint | | The terms in this group occurred between Jan 1, 2016 and Oct 31, 2024 | | |
|  | event relationship | | Any instance of Group 1B occurred within 7 days on or after the first instance of Group 1A | | |
|  | **Group 1B** | | | | |
|  | must have |  | medication | NLM:RXNORM:1596450 | gentamicin |
|  | cannot have |  | medication | NLM:RXNORM:9384 | rifampin |
| Group 2 | | | | | |
|  | **Group 2A** | | | | |
|  | must have |  | diagnosis | UMLS:ICD10CM:T82.6 | Infection and inflammatory reaction due to cardiac valve prosthesis (at least 18 years old at event) |
|  | date constraint | | The terms in this group occurred between Jan 1, 2016 and Oct 31, 2024 | | |
|  | event relationship | | Any instance of Group 2B occurred within 7 days before or up to 7 days after the first instance of Group 2A | | |
|  | **Group 2B** | | | | |
|  | must have | any of | diagnosis | UMLS:ICD10CM:B95.61 | Methicillin susceptible Staphylococcus aureus infection as the cause of diseases classified elsewhere |
|  |  |  | diagnosis | UMLS:ICD10CM:A49.01 | Methicillin susceptible Staphylococcus aureus infection, unspecified site |
|  |  |  | diagnosis | UMLS:ICD10CM:A41.01 | Sepsis due to Methicillin susceptible Staphylococcus aureus |
|  |  |  | diagnosis | UMLS:ICD10CM:B95.62 | Methicillin resistant Staphylococcus aureus infection as the cause of diseases classified elsewhere |
|  |  |  | diagnosis | UMLS:ICD10CM:A49.02 | Methicillin resistant Staphylococcus aureus infection, unspecified site |
|  |  |  | diagnosis | UMLS:ICD10CM:A41.02 | Sepsis due to Methicillin resistant Staphylococcus aureus |
|  |  |  | diagnosis | UMLS:ICD10CM:B95.6 | Staphylococcus aureus as the cause of diseases classified elsewhere |
|  |  |  | diagnosis | UMLS:ICD10CM:A41.0 | Sepsis due to Staphylococcus aureus |
|  |  |  | laboratory | UMLS:LNC:88269-6 | Staphylococcus aureus gyrB gene [Presence] by Probe in Positive blood culture (labResult: Positive) |
|  |  |  | laboratory | UMLS:LNC:92777-2 | Staphylococcus aureus DNA [Presence] by NAA with probe detection in Positive blood culture (labResult: Positive) |
|  |  |  | laboratory | UMLS:LNC:85765-6 | Staphylococcus aureus DNA [Presence] by NAA with non-probe detection in Positive blood culture (labResult: Positive) |

Supplemental Table 3. Participants characteristics before propensity score matching between rifampicin group versus rifampicin plus gentamicin group

|  | Rifampicin group (N=353) | Rifampicin plus gentamicin group (N=651) | p-value |
| --- | --- | --- | --- |
| Age group |  |  |  |
| Aged 18–49 years | 125 (35.4%) | 289 (44.4%) | 0.006 |
| Aged 50–64 years | 77 (21.8%) | 162 (24.9%) | 0.275 |
| Aged 65–79 years | 118 (33.4%) | 175 (26.9%) | 0.029 |
| Aged 80 years or older | 33 (9.3%) | 25 (3.8%) | <0.001 |
| Gender |  |  |  |
| Females | 114 (32.3%) | 214 (32.9%) | 0.852 |
| Race/ethnicity |  |  |  |
| White | 306 (86.7%) | 521 (80.0%) | 0.008 |
| Black or African American | 14 (4.0%) | 64 (9.8%) | 0.001 |
| Asian | <=10 (NA) | 11 (1.7%) | NA |
| Others | <=10 (NA) | 18 (2.8%) | NA |
| Unknown | 22 (6.2%) | 27 (4.1%) | 0.143 |
| Comorbidity |  |  |  |
| Presence of cardiac pacemaker (Z95.0) | 55 (15.6%) | 81 (12.4%) | 0.165 |
| Presence of other cardiac and vascular implants and grafts (Z95.8) | 56 (15.9%) | 100 (15.4%) | 0.834 |
| Mental and behavioral disorders due to psychoactive substance use (F10-19) | 131 (37.1%) | 315 (48.4%) | 0.001 |
| Heart failure (I50) | 199 (56.4%) | 374 (57.5%) | 0.742 |
| Diabetes mellitus (E08-13) | 113 (32.0%) | 189 (29.0%) | 0.326 |
| Certain disorders involving the immune mechanism (D80-89) | 13 (3.7%) | 29 (4.5%) | 0.560 |
| Chronic lower respiratory diseases (J40-4A) | 91 (25.8%) | 192 (29.5%) | 0.212 |
| Diseases of the nervous system (G00-99) | 237 (67.1%) | 494 (75.9%) | 0.003 |
| Acute kidney failure and chronic kidney disease (N17-19) | 237 (67.1%) | 440 (67.6%) | 0.885 |
| Shock, not elsewhere classified (R57) | 66 (18.7%) | 179 (27.5%) | 0.002 |
| Methicillin resistant Staphylococcus aureus infection, unspecified site (A49.02) | 21 (5.9%) | 42 (6.5%) | 0.754 |
| Methicillin resistant Staphylococcus aureus infection as the cause of diseases classified elsewhere (B95.62) | 87 (24.6%) | 170 (26.1%) | 0.611 |
| Sepsis due to Methicillin resistant Staphylococcus aureus (A41.02) | 76 (21.5%) | 135 (20.7%) | 0.769 |
| History of ICU admission | 142 (40.2%) | 326 (50.1%) | 0.003 |
| Administered antimicrobials |  |  |  |
| Cefazolin | 127 (36.0%) | 268 (41.2%) | 0.120 |
| Anti-staphylococcal MSSA) penicillins | 91 (25.8%) | 286 (43.9%) | <0.001 |
| Vancomycin | 198 (56.1%) | 472 (72.5%) | <0.001 |
| Daptomycin | 56 (15.9%) | 57 (8.8%) | 0.001 |
| Linezolid | 14 (4.0%) | 19 (1.5%) | 0.459 |
| Ceftaroline | 26 (7.4%) | 32 (4.9%) | 0.120 |

Abbreviations: ICU, intensive care unit; MSSA, methicillin-sensitive *Staphylococcus aureus*; NA; not assessed.

Supplemental Table 4. Participants characteristics after propensity score matching between rifampicin group versus rifampicin plus gentamicin group

|  | Rifampicin group (N=324) | Rifampicin plus Gentamicin group (N=324) | p-value | Standard difference |
| --- | --- | --- | --- | --- |
| Age group |  |  |  |  |
| Aged 18–49 years | 121 (37.3%) | 109 (33.6%) | 0.325 | 0.077 |
| Aged 50–64 years | 72 (22.2%) | 76 (23.5%) | 0.708 | 0.029 |
| Aged 65–79 years | 106 (32.7%) | 118 (36.4%) | 0.322 | 0.078 |
| Aged 80 years or older | 25 (7.7%) | 21 (6.5%) | 0.541 | 0.048 |
| Gender |  |  |  |  |
| Females | 108 (33.3%) | 101 (31.2%) | 0.556 | 0.046 |
| Race/ethnicity |  |  |  |  |
| White | 279 (86.1%) | 285 (88.0%) | 0.483 | 0.055 |
| Black or African American | 14 (4.3%) | 11 (3.4%) | 0.541 | 0.048 |
| Asian | <=10 (NA) | <=10 (NA) | NA | NA |
| Others | <=10 (NA) | <=10 (NA) | NA | NA |
| Unknown | 20 (6.2%) | 15 (4.6%) | 0.385 | 0.068 |
| Comorbidity |  |  |  |  |
| Presence of cardiac pacemaker (Z95.0) | 51 (15.7%) | 41 (12.7%) | 0.260 | 0.089 |
| Presence of other cardiac and vascular implants and grafts (Z95.8) | 52 (16.0%) | 43 (13.3%) | 0.318 | 0.079 |
| Mental and behavioral disorders due to psychoactive substance use (F10-19) | 129 (39.8%) | 120 (37.0%) | 0.467 | 0.057 |
| Heart failure (I50) | 187 (57.7%) | 181 (55.9%) | 0.634 | 0.037 |
| Diabetes mellitus (E08-13) | 107 (33.0%) | 105 (32.4%) | 0.867 | 0.013 |
| Certain disorders involving the immune mechanism (D80-89) | 11 (3.4%) | 15 (4.6%) | 0.423 | 0.063 |
| Chronic lower respiratory diseases (J40-4A) | 87 (26.9%) | 81 (25.0%) | 0.591 | 0.042 |
| Diseases of the nervous system (G00-99) | 230 (71.0%) | 226 (69.8%) | 0.731 | 0.027 |
| Acute kidney failure and chronic kidney disease (N17-19) | 225 (69.4%) | 222 (68.5%) | 0.799 | 0.020 |
| Shock, not elsewhere classified (R57) | 66 (20.4%) | 63 (19.4%) | 0.768 | 0.023 |
| Methicillin resistant Staphylococcus aureus infection, unspecified site (A49.02) | 19 (5.9%) | 23 (7.1%) | 0.523 | 0.050 |
| Methicillin resistant Staphylococcus aureus infection as the cause of diseases classified elsewhere (B95.62) | 81 (25.0%) | 81 (25.0%) | 1.000 | <0.001 |
| Sepsis due to Methicillin resistant Staphylococcus aureus (A41.02) | 72 (22.2%) | 69 (21.3%) | 0.775 | 0.022 |
| History of ICU admission | 139 (42.9%) | 137 (42.3%) | 0.874 | 0.012 |

Abbreviations: ICU, intensive care unit; NA; not assessed

Comorbidity was based on ICD-10 codes provided on the day of prosthetic valve endocarditis diagnosis or before.

Supplemental Table 5. Participants characteristics after propensity score matching between gentamicin group versus rifampicin plus gentamicin group

|  | Gentamicin group (N=347) | Rifampicin plus Gentamicin group (N=347) | p-value | Standard difference |
| --- | --- | --- | --- | --- |
| Age group |  |  |  |  |
| Aged 18–49 years | 154 (44.4%) | 153 (44.1%) | 0.939 | 0.006 |
| Aged 50–64 years | 90 (25.9%) | 95 (27.4%) | 0.668 | 0.033 |
| Aged 65–79 years | 84 (24.2%) | 82 (23.6%) | 0.859 | 0.014 |
| Aged 80 years or older | 19 (5.5%) | 17 (4.9%) | 0.732 | 0.026 |
| Gender |  |  |  |  |
| Females | 125 (36.0%) | 118 (34.0%) | 0.578 | 0.042 |
| Race/ethnicity |  |  |  |  |
| White | 282 (81.3%) | 284 (81.8%) | 0.845 | 0.015 |
| Black or African American | 38 (11.0%) | 35 (10.1%) | 0.710 | 0.028 |
| Asian | <=10 (NA) | <=10 (NA) | NA | NA |
| Others | <=10 (NA) | <=10 (NA) | NA | NA |
| Unknown | 12 (3.5%) | 15 (4.3%) | 0.556 | 0.045 |
| Comorbidity |  |  |  |  |
| Presence of cardiac pacemaker (Z95.0) | 51 (14.7%) | 51 (14.7%) | 1.000 | <0.001 |
| Presence of other cardiac and vascular implants and grafts (Z95.8) | 56 (16.1%) | 60 (17.3%) | 0.684 | 0.031 |
| Mental and behavioral disorders due to psychoactive substance use (F10-19) | 172 (49.6%) | 178 (51.3%) | 0.649 | 0.035 |
| Heart failure (I50) | 206 (59.4%) | 207 (59.7%) | 0.938 | 0.006 |
| Diabetes mellitus (E08-13) | 106 (30.5%) | 111 (32.0%) | 0.682 | 0.031 |
| Certain disorders involving the immune mechanism (D80-89) | 15 (4.3%) | 23 (6.6%) | 0.182 | 0.101 |
| Chronic lower respiratory diseases (J40-4A) | 108 (31.1%) | 98 (28.2%) | 0.406 | 0.063 |
| Diseases of the nervous system (G00-99) | 264 (76.1%) | 266 (76.7%) | 0.858 | 0.014 |
| Acute kidney failure and chronic kidney disease (N17-19) | 256 (73.8%) | 251 (72.3%) | 0.669 | 0.032 |
| Shock, not elsewhere classified (R57) | 91 (26.2%) | 98 (28.2%) | 0.551 | 0.045 |
| Methicillin resistant Staphylococcus aureus infection, unspecified site (A49.02) | 24 (6.9%) | 24 (6.9%) | 1.000 | <0.001 |
| Methicillin resistant Staphylococcus aureus infection as the cause of diseases classified elsewhere (B95.62) | 89 (25.6%) | 91 (26.2%) | 0.862 | 0.013 |
| Sepsis due to Methicillin resistant Staphylococcus aureus (A41.02) | 84 (24.2%) | 80 (23.1%) | 0.721 | 0.027 |
| History of ICU admission | 183 (52.7%) | 184 (53.0%) | 0.939 | 0.006 |

Abbreviations: ICU, intensive care unit; NA; not assessed

Comorbidity was based on ICD-10 codes provided on the day of prosthetic valve endocarditis diagnosis or before.

Supplemental Figure 1. Survival curve in rifampicin group versus rifampicin plus gentamicin group since diagnosis (index event)


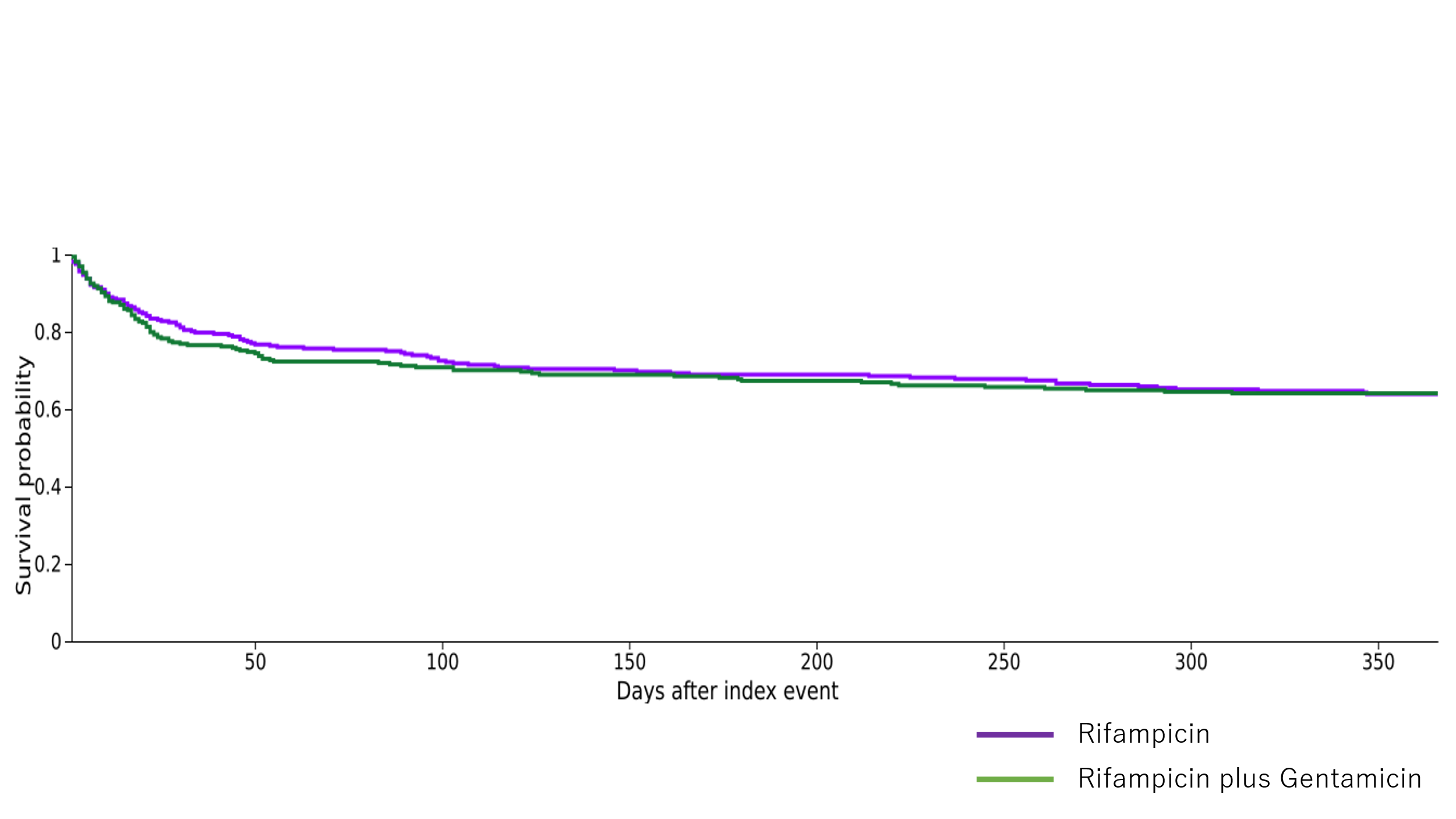


Supplemental Figure 2. Survival curve in gentamicin group versus rifampicin plus gentamicin group since diagnosis (index event)


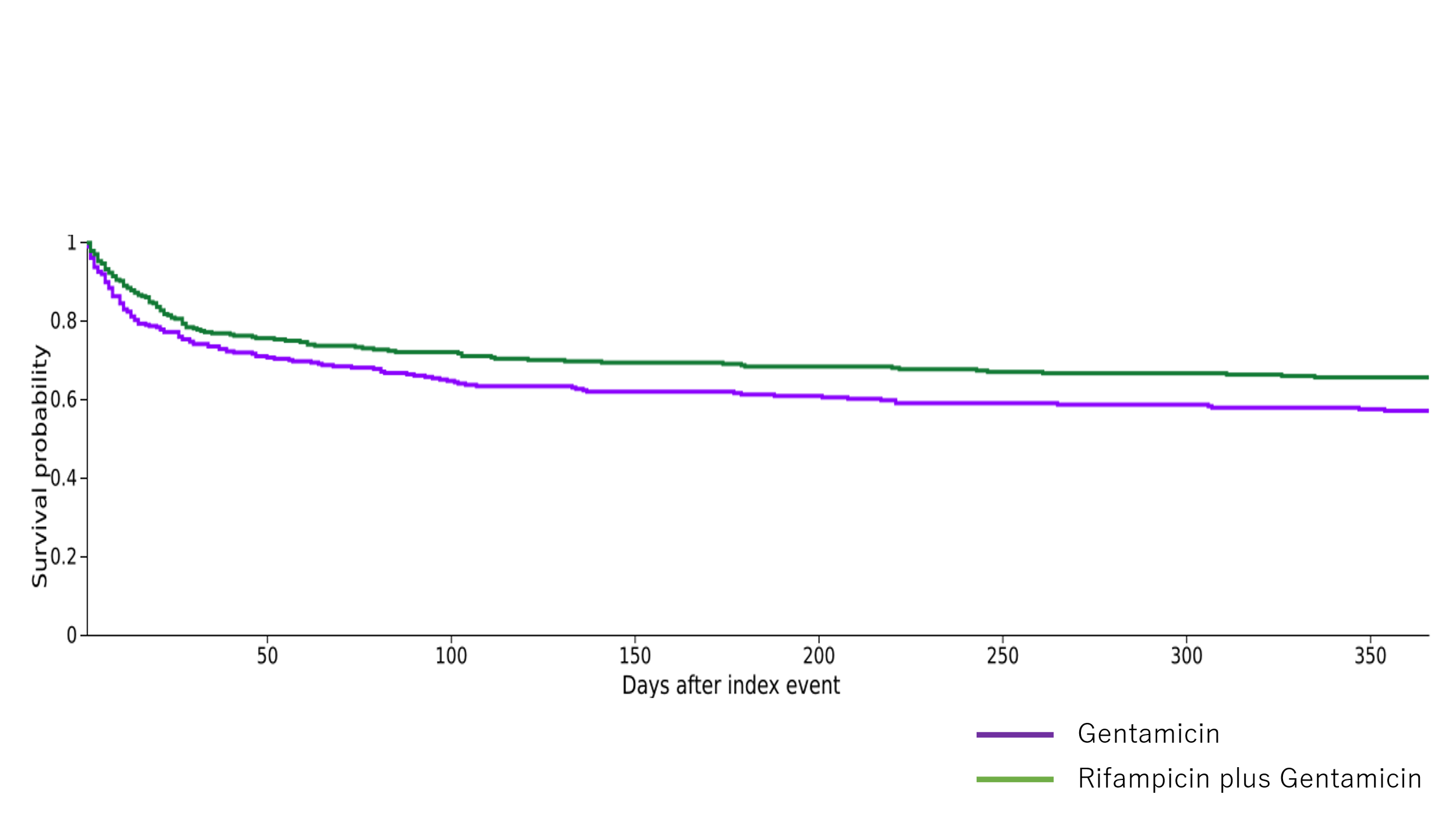

Supplement: dlaf246_Supplementary_Data [file dlaf246_supplementary_data.docx]
